# Optimum Production Conditions, Purification, Identification, and Antioxidant Activity of Violaxanthin from Microalga *Eustigmatos* cf. *polyphem* (Eustigmatophyceae)

**DOI:** 10.3390/md16060190

**Published:** 2018-06-01

**Authors:** Feifei Wang, Luodong Huang, Baoyan Gao, Chengwu Zhang

**Affiliations:** Institute of Hydrobiology, Department of Ecology, Jinan University, Guangzhou 510632, China; feifei2013360@126.com (F.W.); hld8124@sina.com (L.H.); gaobaoyan1211@126.com (B.G.)

**Keywords:** *Eustigmatos* cf. *polyphem*, violaxanthin, culture conditions, purification, antioxidant activity

## Abstract

Violaxanthin is a major xanthophyll pigment in the microalga *Eustigmatos* cf. *polyphem*, but the amount produced after propagation can vary depending upon culture conditions. In this study, the effects of cultivation time, nitrogen concentration, light intensity, and culture mode on violaxanthin production were investigated. The results showed that this microalga vigorously grew and maintained a high level of violaxanthin in the fed-batch culture, and the highest violaxanthin productivity of 1.10 ± 0.03 mg L^−1^ d^−1^ was obtained under low light illumination with 18 mM of initial nitrogen supply for ten days. Additionally, violaxanthin was purified from *E.* cf. *polyphem* by silica gel chromatography and preparative high-performance liquid chromatography (PHPLC), and identified with high-resolution mass spectrometry (HRMS). The antioxidant activity of the purified violaxanthin was evaluated by three tests in vitro: reducing power assay, 2,2-diphenyl-1-picrylhydrazyl (DPPH), and 2,2-azobis-3-ethylbenzthiazoline-6-sulphonic acid (ABTS) radical assays. The strongest inhibition of purified violaxanthin occurred during the scavenging of ABTS^+^ radicals, with EC_50_ of 15.25 μg mL^−1^. In conclusion, this is the first report to investigate the effects of different culture conditions on violaxanthin accumulation in *E.* cf. *polyphem* and provide a novel source for the production of violaxanthin that can be used for food and pharmaceutical applications.

## 1. Introduction

Carotenoids belong to the isoprenoid group that is commonly characterized by a C_40_ tetraterpenoid structure built from eight C_5_ isoprenoid units [1]. More than 750 carotenoids have been isolated from natural sources, and these carotenoids were divided into two different groups according to their chemical structures: (1) hydrocarbon carotenoids, generally named carotenes, which consist of only carbon and hydrogen, and (2) oxygenated carotenoids, which are called xanthophylls [2]. Carotenoids are rich in unsaturated groups, and many studies have confirmed that they exhibit strong activity in the scavenging of reactive oxygen species (ROS) [3,4]. Traditionally, carotenoids have been used as colorants in the food and feed industries. Carotenoids have also been formulated as dietary antioxidants, and extensive health-promoting characteristics, such as antioxidative, anti-arteriosclerosis, antiproliferative, and anticancer effects, as well as vision protection [5,6,7]. Since these protective functions were thought to be attributed to their strong antioxidant properties [8], various carotenoids were isolated and purified from natural sources for the evaluation of their potential antioxidant activity.

Microalgae contain abundant quantities of natural antioxidants, such as carotenoids [7,9]. In microalgae, the carotenoids usually function as accessory pigments and structural components of light-harvesting complexes in photosystems, and participate in photosynthetic reactions [10]. In addition, some carotenoids can serve as photoprotective agents to protect the photosynthetic apparatus from excess light damage by scavenging ROS, such as singlet oxygen and free radicals, and be responsible for phototaxis [11]. Various microalgae can accumulate large amounts of carotenoids, such as fucoxanthin, astaxanthin, lutein, canthaxanthin, and β-carotene, some of which have been commercialized, including β-carotene from the halophile green microalga *Dunaliella salina*, astaxanthin from the freshwater green microalga *Haematococcus lacustris* (formerly *Haematococcus pluvialis*), fucoxanthin from diatoms, and lutein from some Chlorophycean microalgae (class Chlorophyceae) [10,12]. At present, these main microalgal carotenoids have been used as colorants in food and nutraceuticals in human health care.

Apart from the above-mentioned primary carotenoids, microalgae may also accumulate other important carotenoids, such as violaxanthin and canthaxanthin. Violaxanthin is a natural xanthophyll pigment that is orange-colored. It is biosynthesized from zeaxanthin by epoxidation and has double 5,6-epoxy groups, which are found in orange-colored fruits, green vegetables, and microalgae [13,14]. Fu et al. [2] reported that violaxanthin purified from water spinach/water morning glory (*Ipomoea aquatica*, class Dicotyledonae) has a strong ABTS^+^ radical scavenging activity, and it also demonstrated valid inhibition of lipid peroxidation and red blood cell hemolysis [2]. These actions indicated that violaxanthin has the potential to be widely applied in medical and health products. Among microalgal sources, there are only two studies reporting violaxanthin isolation; one described the isolation from the green microalga *Dunaliella tertiolecta* [15], and the other used *Chloroidium ellipsoideum* (formerly *Chlorella ellipsoidea*) [10] as the source. The violaxanthin purified from these two microalgae also exhibited anti-proliferative, anti-inflammatory, and proapoptotic activity against human cancer cell lines in vitro [16]. There are still additional microalgae resources that could be utilized, thus broadening the sources and new functional applications of violaxanthin.

*Eustigmatos* cf. *polyphem* (formerly determined as *Myrmecia bisecta*) is a yellow-green unicellular edaphic microalga belonging to the class Eustigmatophyceae [17]. Our previous work found that *E.* cf. *polyphem* can accumulate large quantities of lipids and β-carotene under stress conditions [18,19], and because of its high lipid productivity production, its photosynthetic apparatus is worthy of closer examination. The eustigmatophycean microalgae lack chlorophylls *b* and *c*, and they mainly contain chlorophyll *a*, violaxanthin, vaucheriaxanthin ester, and β-carotene as photosynthetic pigments [20]. Violaxanthin is the dominant carotenoid that usually combines with chlorophyll *a* and apoprotein to form violaxanthin-chorophyll-*a*-binding protein (VCP) complexes in the thylakoids of chloroplast, and takes part in light harvesting [21,22]. Violaxanthin is a structural component of the xanthophyll cycle that protects the photosynthetic apparatus against an excess of light via non-photochemical fluorescence quenching [23]. Demmig-Adams et al. [24] proved that the xanthophyll cycle is involved in light protection, and the amount of violaxanthin in microalgae varies with the light intensity. Pasquet et al. [15] reported that the low violaxanthin production in *Dunaliella tertiolecta* restricts its therapeutic applications, but the effects of culture conditions on violaxanthin accumulation in microalgae have not yet been investigated.

The content of violaxanthin in microalgae is variable, because it is affected by the external environment [25,26]. In the present study, the effects of different culture conditions on violaxanthin production in *E.* cf. *polyphem*, including culture time, initial nitrogen concentration in culture medium, light intensity, and culture mode, were investigated. Additionally, the violaxanthin was extracted, purified, and identified from the algal biomass, and its antioxidant activity was evaluated in vitro. This work is aimed at providing another new microalgal source of violaxanthin and maximizing the violaxanthin production in *E.* cf. *polyphem* for potential applications in food and pharmaceuticals.

## 2. Results and Discussion

### 2.1. Effect of Cultivation Time on Violaxanthin Production

**Figure 1:**
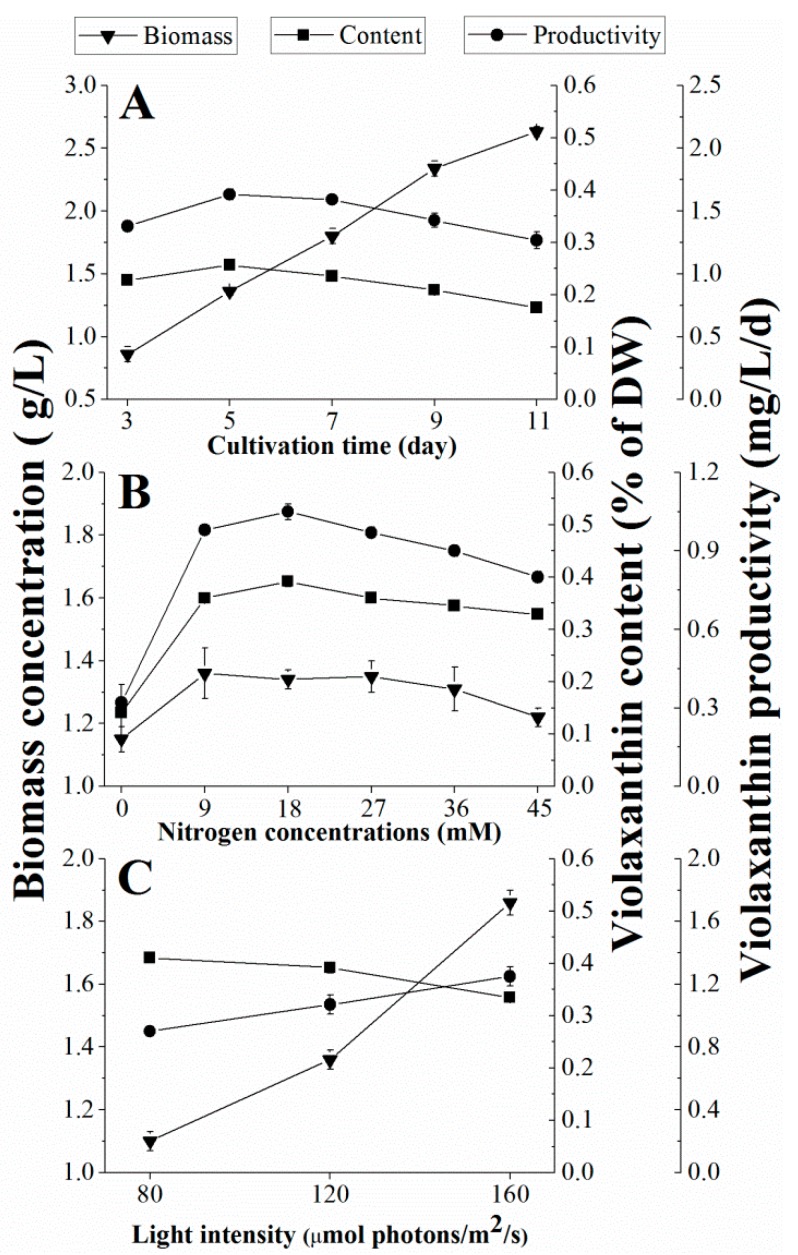
Effects of (**A**) cultivation time, (**B**) nitrogen concentration, and (**C**) light intensity on the violaxanthin production of *Eustigmatos* cf. *polyphem*. (**A** and **B**) The algae were cultured at a low light of 120 μmol photons m^−2^ s^−1^. Values are expressed as the mean ± SD from three replicates; DW, dry weight.

The effect of cultivation time on violaxanthin production in *E.* cf. *polyphem* is shown in Figure 1A. The algal biomass concentration increased with the prolonging of culture time, and it reached 2.63 ± 0.04 g L^−1^ on the 11th day with low light illumination. However, the content of violaxanthin in *E.* cf. *polyphem* increased to the highest level of 0.39% (of DW) on the fifth day, and then gradually decreased to 0.30% on the 11th day. This result was in agreement with the reports of Sobrino et al. [27], who found that the violaxanthin content decreased with culture time in *Nannochloropsis* strains (class Eustigmatophyceae). Li et al. [18] reported that the violaxanthin content in *E.* cf. *polyphem* quickly decreased with prolonged culture time under high light illumination, while it decreased at a relatively slow rate in this study. Low light intensity was used in our study, which might slow the degradation of violaxanthin.

Violaxanthin productivity is a suitable evaluation parameter for violaxanthin production due to the contradiction between algal biomass and violaxanthin content. The highest violaxanthin productivity of 1.07 ± 0.03 mg L^−1^ d^−1^ was obtained on the fifth day (Figure 1A) and, therefore, five days of culture time was selected and used for the next study.

### 2.2. Effect of Nitrogen Concentration on Violaxanthin Production

Nitrogen supply is essential for all microalgae growth, and its redundancy or limitation influences the biomass and biochemical composition of microalgae [28]. Therefore, the initial nitrogen concentration (0, 9, 18, 27, 36, and 45 mM) was designed to investigate its effect on violaxanthin production. As Figure 1B shows, the lowest nitrogen concentration group (LNCG, 0 mM) and the highest nitrogen concentration group (HNCG, 45 mM) adversely affected the microalgae growth and violaxanthin accumulation, especially the LNCG. The lowest biomass, violaxanthin content, and violaxanthin productivity, which were 1.15 ± 0.04 g L^−1^, 0.14% ± 0.05 (of DW), and 0.32 ± 0.01 mg L^−1^ d^−1^, respectively, were obtained at this nitrogen concentration (0 mM). The violaxanthin content increased with the higher nitrogen concentrations when the initial nitrogen concentration was between 0 mM and 18 mM. These results were consistent with the report of Gao et al. [26]. Then, the violaxanthin content gradually decreased when the initial nitrogen concentration ≥18 mM (Figure 1B). These results indicated that very high or insufficient nitrogen concentrations were disadvantageous to both algal growth and violaxanthin accumulation. In addition, the highest violaxanthin productivity (1.05 ± 0.03 mg L^−1^ d^−1^) was achieved at 18 mM of the initial nitrogen despite a biomass concentration that was lower than 9 mM. Thus, 18 mM initial nitrogen concentration was shown to be more conducive to violaxanthin accumulation than the other investigated concentrations.

### 2.3. Effect of Light Intensity on Violaxanthin Production

Violaxanthin often exists in photosynthetic apparatus, and it is sensitive to light. In the xanthophyll cycle, violaxanthin transforms into zeaxanthin by de-epoxidation under high light, but zeaxanthin is epoxidated to violaxanthin under low light illumination [24]. Therefore, the amount of violaxanthin varies with the light intensity. In this study, three different light intensities of 80, 120, and 160 μmol photons m^−2^ s^−1^ were introduced to study the effect on violaxanthin production (Figure 1C).

As expected, the biomass accumulation was correlated with increasing light intensity, but the content of violaxanthin decreased with increasing light intensity, which was consistent with a previously published study [27]. Under the light intensity of 160 μmol photons m^−2^ s^−1^, the highest biomass was 1.86 ± 0.05 mg L^−1^ with a high violaxanthin productivity of 1.25 ± 0.06 mg L^−1^ d^−1^, but the violaxanthin content (0.33 ± 0.01% (of DW)) was significantly lower than that obtained at the light intensity of 80 and 120 μmol photons m^−2^ s^−1^ (*p* < 0.05). Moreover, we found that the violaxanthin content under high light illumination (160 μmol photons m^−2^ s^−1^) quickly decreased beyond five days cultivation time in the preliminary experiment. Therefore, the lower light intensity was more suitable for the accumulation of violaxanthin, and 120 μmol photons m^−2^ s^−1^ was selected as the appropriate light intensity.

### 2.4. Effect of Culture Mode on Violaxanthin Production

As described earlier, the violaxanthin content in *E.* cf. *polyphem* was affected by the external environment and, therefore, fed-batch culture was used to overcome the effects of nutrient shortages and extracellular metabolite inhibition.

The effect of culture mode on violaxanthin production is presented in Table 1. In contrast with the control (the batch culture), the microalgae vigorously grew in the fed-batch culture, and the highest biomass (5.15 ± 0.03 g L^−1^) was obtained on day 20. Moreover, as the culture time increased, the violaxanthin content was kept at a high level in the fed-batch culture and was significantly higher than that of the control. Although the violaxanthin productivity decreased with prolonged time in the fed-batch culture, it was still higher than that of the control, and the descent rate was much slower. Accordingly, the fed-batch culture was more suitable for violaxanthin production.

### 2.5. Purification and Identification of Violaxanthin

**Figure 2:**
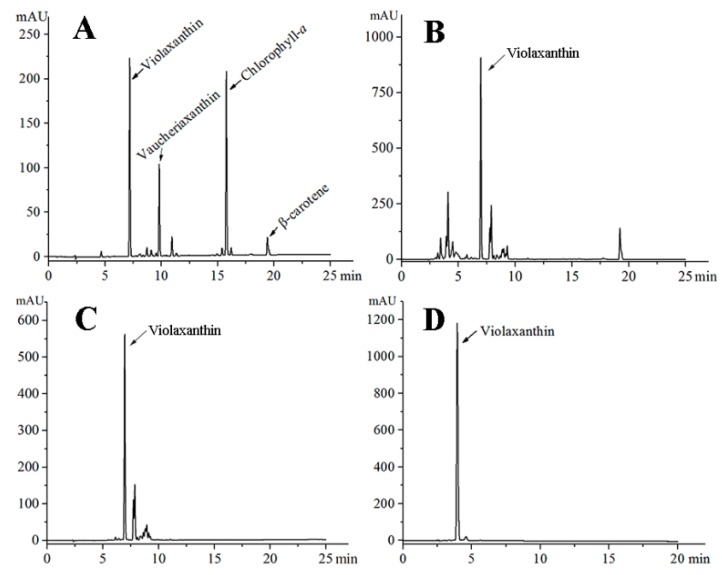
HPLC analysis of the pigment profile of the targeted fraction in each isolation and purification procedure. (**A**) ethanol extract; (**B**) saponification; (**C**) silica gel column chromatography; and (**D**) preparative HPLC.

Violaxanthin was extracted, isolated, and purified following the procedures illustrated in Figure 2. The pigment profile of the ethanol extract from *E.* cf. *polyphem* was obtained by high-performance liquid chromatography (HPLC). The result showed that violaxanthin, vaucheriaxanthin, chlorophyll *a*, and β-carotene were the major pigments (Figure 2A), which was consistent with the reports of Gao et al. [26]. Figure 2B showed that, after saponification, the peak of chlorophyll *a* was not detected, and this indicated that chlorophyll *a* was successfully removed. The concentrated ethyl acetate extract (2.77 g) was separated using silica gel column chromatography to obtain FB_2_ (136 mg), and the purity of violaxanthin in FB_2_ reached 62% (Figure 2C) based on the HPLC analysis. Further purification was performed using PHPLC, and FB_2-2_ (10.0 mg) showed a single peak in the HPLC chromatogram at a purity ≥ 95% (Figure 2D).

**Figure 3:**
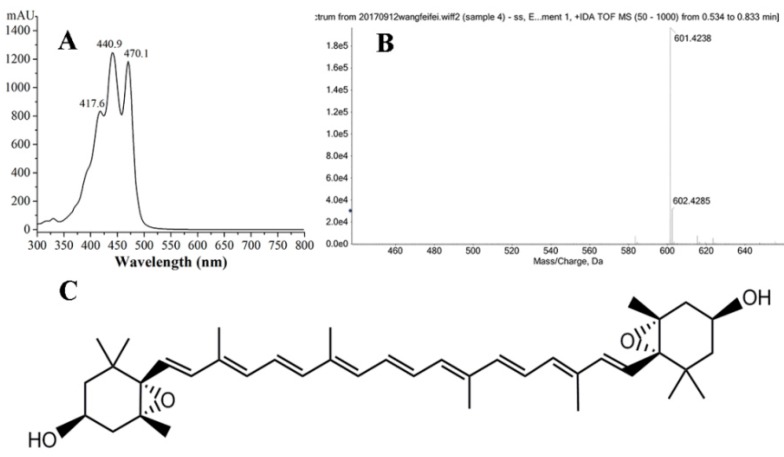
Identification of the purified violaxanthin from *Eustigmatos* cf. *polyphem*. (**A**) UV–VIS spectrum; (**B**) mass spectrum of violaxanthin; and (**C**) the chemical structure of violaxanthin.

Violaxanthin was then identified from FB_2-2_ based on physicochemical properties, absorption spectra, and HRMS spectrum. The dried FB_2-2_ powder was orange red, and it showed a characteristic UV–VIS spectrum. The maximum absorption peaks at 417.6 nm, 440.9 nm, and 470.1 nm in Figure 3A, were in agreement with those of previously published studies [10,15]. In addition, the HRMS spectrum showed two characteristic fragment ion peaks at *m*/*z* 601.4238 [M + H]^+^ and *m*/*z* 602.4285 [M + 2H]^+^, which corresponded to the molecular weight of the purified violaxanthin at 600.4 (Figure 3B). The results were consistent with previously published reports [2,15]. Therefore, this purified pigment was distinctly identified as 5,6,5′,6′-diepoxy-5,6,5′,6′-tetrahydro-β,β-carotene-3,3′-diol (Figure 3C).

### 2.6. Reducing Power of Purified Violaxanthin

**Figure 4:**
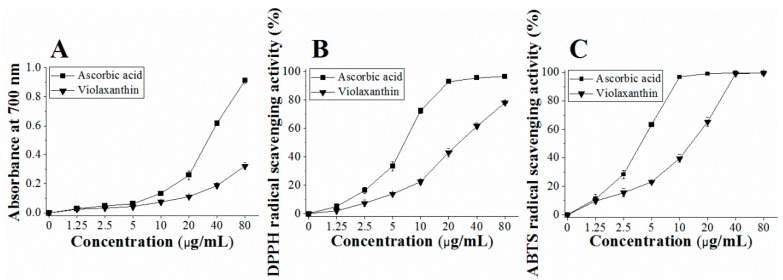
Antioxidant activity assays for purified violaxanthin from *Eustigmatos* cf. *polyphem*. (**A**) Reducing power; (**B**) scavenging of DPPH radicals; and (**C**) scavenging of ABTS radical. Ascorbic acid was used as a positive control. Values are shown as the mean ± standard deviation from three independent experiments.

The test sample with strong reducing power could serve as an electron donor to produce stable products and achieve the effect of scavenging radicals, and therefore the reducing power is an important indicator of the evaluation of antioxidant capacity [29]. In this study, the reducing power was determined by Fe^3+^-Fe^2+^ transformation in the presence of different concentrations of violaxanthin. The Fe^2+^ was monitored by measuring the formation of Perl’s Prussian blue at 700 nm, and the results are shown in Figure 4A. It was discernible that the reducing power of the purified violaxanthin and the positive control (ascorbic acid) increased in a concentration-dependent manner, but the purified violaxanthin showed weak reducing power in contrast with ascorbic acid. At 80 μg mL^−1^, purified violaxanthin exhibited a reducing power of 0.32 ± 0.02 abs, while the reducing power of ascorbic acid reached 0.91 ± 0.02 abs. Additionally, we found that the reducing power of violaxanthin and ascorbic acid was low at 1.25 to 5 μg mL^−1^ with no significant difference, but the reducing power of ascorbic acid notably increased when the concentration of ascorbic acid was greater than 10 μg mL^−1^.

### 2.7. DPPH Radical-Scavenging Activity of Purified Violaxanthin

The DPPH radical assay is one of the most widely used methods for testing the antioxidant activity of various compounds [30]. DPPH is a stable nitrogen-centered radical, that is violet colored in ethanol and has a strong absorption peak at 517 nm. Therefore, this assay is based on the measurement of the absorbance change of the reaction solution at 517 nm after violaxanthin was mixed with DPPH, and the results are shown in Figure 4B. The DPPH-radical scavenging activity of purified violaxanthin was concentration-dependent. When the concentration was varied from 1.25–80 μg mL^−1^, the inhibition percentage of purified violaxanthin ranged from 2.15% to 78.17%. Zhang et al. [31] reported that several natural pigments (lutein, lycopene, betalain, and capsanthin) showed an inhibition percentage of 25–60% in the scavenging of DPPH radicals, which were lower than the the inhibition activity of purified violaxanthin at the same concentration. Purified violaxanthin scavenged 50% of DPPH radicals with EC_50_ of 41.42 μg mL^−1^, which was three times smaller compared to fucoxanthin isolated from *Odontella aurita* (phylum Bacillariophyta) (EC_50_ = 140 mg mL^−1^ [32]). Although the scavenging capacity of purified violaxanthin was inferior to the positive control (ascorbic acid), it always exhibited potent potential in the scavenging of DPPH radicals.

### 2.8. ABTS Radical Scavenging Activity of the Purified Violaxanthin

ABTS produces the stable glaucous cation radicals (ABST^+^) by reacting with K_2_(SO_4_)_2_, which has a characteristic peak absorbance at 734 nm. The absorbance of the reaction solution decreased from adding the antioxidants, which were used to assess its antioxidant activity.

As demonstrated in Figure 4C, purified violaxanthin and ascorbic acid both showed strong scavenging activities for ABTS^+^ radicals. When the concentration ≥ 40 μg mL^−1^, purified violaxanthin and ascorbic acid had the same ABTS^+^ scavenging power, with the ability to almost completely scavenged ABTS^+^ radicals. Between the concentrations of 1.25 and 40 μg mL^−1^, the inhibition effect of the purified violaxanthin fortified with increasing concentration, and it showed an inhibition of 99.08 ± 0.32% at 40 μg mL^−1^. The EC_50_ value of the purified violaxanthin was 15.25 μg mL^−1^, and it clearly indicated that violaxanthin has a strong inhibitory effect on ABTS^+^ radicals in comparison with that of fucoxanthin (EC_50_ = 30 μg/mL [32]). Fu et al. [2] reported that violaxanthin purified from water spinach scavenged ABTS^+^ radicals more efficiently than the β-carotenoid and lutein, and that violaxanthin also inhibited a larger quantity of ABTS^+^ radicals as compared to DPPH radicals. These findings were in accordance with our results.

## 3. Materials and Methods

### 3.1. Chemicals and Reagents

Pigment standards including chlorophyll *a*, violaxanthin, vaucheriaxanthin, and β-carotene were purchased from Sigma-Aldrich Chemical Co. (Shanghai, China; http://www.sigmaaldrich.com). Silica gel (200–300 mesh) was obtained from Qing Dao Marine Chemical Co. (Qingdao, China). HPLC-grade solvents used for HPLC analysis were purchased from Guangzhou Runhao Biotech Co. (Guangzhou, China), such as methanol, acetonitrile, acetic ether, and dichloromethane. Other analytical solvents (n-hexane, methanol, and acetone) used in extraction and isolation of violaxanthin were purchased from Guangzhou Runhao Biotech Co. (Guangzhou, China). Deionized water was prepared by a Milli-Q water purification system (Millipore Corp., Bedford, MA, USA).

Chemicals used for their antioxidant activity including 2,2-diphenyl-2-picrylhydrazyl hydrate (DPPH), 2,2′-azino-bis(3-ethylbenzothiazoline-6-sulfonic acid) (ABTS), potassium persulfate, ferrous chloride, potassium ferricyanide, trichloroacetic acid, hydrogen peroxide, ascorbic acid, sodium dihydrogen phosphate, and disodium hydrogen phosphate were obtained from Sangon Biotech Co. (Shanghai, China).

### 3.2. General Analytical Methods

For biomass measurement, 5.0 mL of algal cultures was filtered through a pre-weighed 0.45 μm GF/B filter paper. Then, the filter paper was dried in an oven at 105 °C overnight. The biomass (DW, g/L) was determined by the difference of the weight of the filter paper with microalgal cells (W_2_) and the weight of filter paper (W_1_) and was calculated as (W_2_ – W_1_) × 200.

For pigment profile analysis, 10.0 mg of freeze-dried microalgal powder mixed with 5 mL of methanol was extracted with a magnetic stirrer at 4 °C overnight until the algal residue was colorless. A certain volume of the pigment extracts was obtained by centrifugation (1940× *g*, 10 min) and filtration (Millipore, 0.45 μm), and analyzed by HPLC. The HPLC was conducted on a Dionex model U-3000 instrument equipped with a TC-C18 column (5 μm, 4.6 mm × 250 mm, Agilent, Santa Clara, CA, USA) and a UV–VIS detector (Waters 2998) at 445 nm. The mobile phase used for HPLC was a binary gradient solvent system (solvent A, acetonitrile/water = 9:1; solvent B, ethyl acetate) with a flow rate of 1 mL min^−1^, and the elution program was as follows: 0 min, 100% A, 0% B; 20 min, 100% B, 0% A; 25 min, 100% B, 0% A; 27 min, 100% A, 0% B; 30 min, 100% A, 0% B.

A stock solution of the violaxanthin standard was prepared in methanol (HPLC grade), and then serially diluted to a final concentration of 60, 30, 15, 7.5, 3.75, and 1.875 μg mL^−1^. A standard curve (y (μg mL^−1^) = 3110.60x − 5.98, *R*^2^ = 0.999) of violaxanthin was drawn on the basis of these concentrations by HPLC. Thus, the violaxanthin production in the *E.* cf. *polyphem* biomass was quantified by the following equations:

(1) $$\mathrm{Violaxanthin}\;\mathrm{content}\;\left(\mathrm{VC},\;\%\;\mathrm{of}\;\mathrm{DW}\right)\;=\;\frac{{C\;\times \;V\;\times \;10}^{-3}}{m}\;\times \;100$$

(2) $$\mathrm{Violaxanthin}\;\mathrm{productivity}\;\left({\mathrm{mg}\;L}^{-1}d^{-1}\right)\;=\;\frac{{\mathrm{DW}\;\times \;\mathrm{VC}\%\;\times \;10}^{3}}{t}$$

where m (mg) denotes the weight of microalgal powder, V (mL) denotes the volume of the extraction solvent; DW denotes the dry weight of biomass (g L^−1^); t (day) denotes the cultivation time, and C (μg mL^−1^) denotes the concentration of the extracted violaxanthin as determined by HPLC analysis.

### 3.3. Microalgae and Cultivation Conditions

The microalga *Eustigmatos* cf. *polyphem* (CAUP-H4302, formerly determined as *Myrmecia bisecta*) used in this study was obtained from the CAUP Culture Collection of Algae. *E.* cf. *polyphem* cells were maintained in a modified BG-11 (mBG-11) medium [26] and deposited in our laboratory. The inocula were prepared by culturing the microalgae in a bubble column glass photobioreactor (Ø6 × 60 cm) with 1.2 L of mBG-11 medium, and growing under continuous 60–70 μmol photons m^−2^ s^−1^ of white fluorescent light illumination at 25 ± 1 °C for 7–9 days. The seed cultures were then inoculated into the fresh mBG-11 medium for cultivation.

In order to investigate the effects of culture conditions on violaxanthin production in *E.* cf. *polyphem*, several important influencing factors were assessed, including culture time, nitrogen concentration, light intensity, and culture mode. The variation of biomass, violaxanthin content, and violaxanthin productivity were used to evaluate the effects of these factors on violaxanthin production in *E.* cf. *polyphem*. The detailed experimental design was as follows: (1) different cultivation times of 3, 5, 7, 9, and 11 days were set to culture the algal cells at a low light intensity of 120 μmol photons m^−2^ s^−1^; (2) sodium nitrate was used as the nitrogen source, and different initial nitrogen supplementation levels (0, 9, 18, 27, 36, and 45 mM) were designed for the cultivation of *E.* cf. *polyphem* at a low light intensity of 120 μmol photons m^−2^ s^−1^; (3) different light intensities of 80, 120, and 160 μmol photons m^−2^ s^−1^ were exploited to culture *E.* cf. *polyphem* with 18 mM of initial nitrogen supply; (4) *E.* cf. *polyphem* was grown in batch culture or fed-batch culture with low light irradiation of 120 μmol photons m^−2^ s^−1^, and mBG-11medium was replaced at five-day intervals at the beginning of the fifth day in the fed-batch culture. All cultures were inoculated into Ø6 × 60 cm bubble column glass photobioreactors at the same initial cell density of 0.25–0.30 g/L and bubbled with 1% CO_2_ (*v*/*v*) from the bottom of the column. The light intensity was measured with dual radiation meter (DRM-FQ, Apogee Instruments, Inc., Logan, UT, USA). Every experimental group was a set of three replicates.

At the end of the cultivation time, culture samples were harvested by centrifugation at 1940× *g* for 5 min, and then lyophilized in a vacuum freeze-dryer (Christ, Germany). The algal powder was stored at 4 °C prior to analysis.

### 3.4. Extraction, Isolation and Purification of Violaxanthin

**Figure 5:**
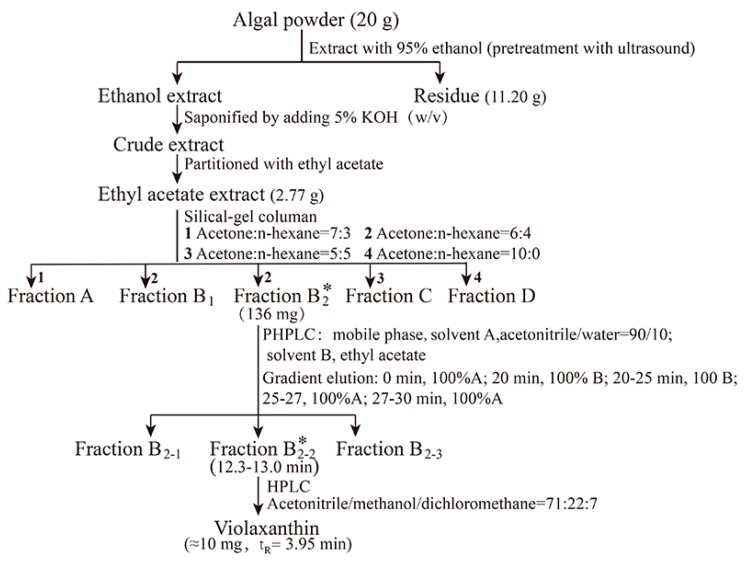
Flowchart of extraction, isolation, and identification of violaxanthin from *Eustigmatos* cf. *polyphem*. “*”, denotes the fraction containing the highest violaxanthin content from the same row.

The extraction, isolation and purification procedures for violaxanthin from *E.* cf. *polyphem* are shown in Figure 5. HPLC was used to analyze the pigment profile in each extraction and isolation procedure. All the processes were performed under weak light.

Freeze-dried microalgal powder (20 g, 0.38% of violaxanthin in dry weight) was mixed with 500 mL of 95% ethanol and subjected to ultrasound-assisted pretreatment in an ultrasonic cleaning bath for 2 h. After ultrasound treatment, the mixture was extracted with a magnetic stirrer in a 40 °C water bath for 4 h. This extraction procedure was repeated four times until the algal powder became colorless. The combined pigment extracts were collected by centrifugation at 1940× *g* for 10 min, and then saponified by adding 5% KOH (*w*/*v*, 100 g) at 50 °C for 30 min under continuous shaking. After the ethanol was removed using a rotary vacuum evaporator at 40 °C, the ethanol extract was re-dissolved in 300 mL of deionized water and partitioned with 400 mL of ethyl acetate in a separatory funnel (1 L) three times. The upper phases were combined and washed with deionized water several times until the KOH was completely removed. The concentrated ethyl acetate extract was obtained by evaporating, and then subjected to a silica-gel column (3 × 40 cm), which was continuously eluted with a stepwise gradient of n-hexane/acetone eluent (*v*/*v* = 7:3, 6:4, 5:5, 0:10) to obtain five fractions (FA, FB_1_, FB_2_, FC, and FD).

According to the HPLC analysis of pigment profiles from the five fractions, FB_2_ was rich in violaxanthin, and was further separated by PHPLC to collect three fractions (FB_2-1_, FB_2-2_, and FB_2-3_). PHPLC was conducted on an Agilent 1100 series instrument (Agilent, Santa Clara, CA, USA) equipped with a TC-C18 column (5 μm, 20 × 250 mm, Agilent, Santa Clara, CA, USA), and the mobile phase was the same as that used for HPLC analysis. The flow rate was 8 mL min^−1^, and ultraviolet detection was a 445 nm. The target fraction (FB_2-2_) was tracked and collected on the basis of the spectral characteristics of the standard.

### 3.5. HPLC Analysis and Identification of Purified Violaxanthin

Purity detection of the targeted fraction (FB_2-2_, 15 μL) was performed using HPLC, and was applied with an isocratic mobile phase of acetonitrile/methanol/dichloromethane (71:22:7, *v*/*v*/*v*) at a flow rate of 1 mL min^−1^; the other details have been previously described. The FB_2-2_ powder was dissolved in methanol, and the accurate molecular weight was determined by high resolution mass spectrum (HRMS, SCIEX, X500R QTOF, Redwood city, CA, USA) using an instrument equipped with an electrospray ionization (ESI) source. Positive mode electrospray ionization ((+) ESI) was conducted to analyze the mass spectra over a mass range of 50–1000 *m*/*z* using a 0.1 unit step size.

### 3.6. Antioxidant Activity Assay

The antioxidant activity of purified violaxanthin was assessed in vitro by three tests, including the reducing power, DPPH radical scavenging activity, and ABTS^+^ radical scavenging activity.

A stock solution (1.0 mg L^−1^) of purified violaxantin was dissolved in ethanol, and then serially diluted with ethanol to a final concentration of 80, 40, 20, 10, 2.5, and 1.25 μg mL^−1^, respectively. Ascorbic acid was used as the positive control, and it was dissolved in deionized water and prepared in a solution of the same concentration as violaxantin. The concentration (μg mL^−1^) of a compound required to scavenge 50% of the radicals is expressed as EC_50_ for the evaluation of potency. All tests were run in triplicate, deionized water was used as a blank control, and the absorbance of the reaction solution was measured with an ultraviolet spectrophotometer (Thermo, Waltham, MA, USA).

Reducing power was determined using a method described by Xia et al. [32]. Briefly, 1.0 mL of different concentrations of purified violaxanthin (1.25–80 μg mL^−1^) was mixed with 0.2 mL of phosphate buffered saline (PBS, 0.2 M, Ph = 6.6) and 1.5 mL of potassium ferricyanide (1%, *w*/*v*), respectively, and the mixture was incubated at 50 °C in a water bath for 20 min. Then, 1 mL of trichloroacetic acid (10%, *w*/*v*) was added to the mixture to quench the reaction. After centrifugation (1940× *g*, 10 min), 2 mL of the supernatant was diluted with 3 mL of deionized water and then reacted with 0.5 mL of ferric chloride (0.5%, *w*/*v*) for 6 min, and the absorbance was rapidly measured at 700 nm. A higher absorbance (A_700_) of the reaction mixture indicated a stronger reducing power of violaxanthin.

DPPH radical scavenging activity of purified violaxanthin was evaluated according to the method of Bai et al. [33] with a slight modification. Two milliliters of DPPH methanolical solution (0.1 mM) was added to 1 mL of different concentrations of the purified violaxanthin (1.25–80 μg mL^−1^). The mixture was vigorously shaken, and then maintained at room temperature in the dark for 30 min. The absorbance of the reaction solution was rapidly measured at 517 nm, and the scavenging ability was calculated using the following equation: DPPH radical scavenging activity (%) = [(A_control_ − A_test_)/A_control_] × 100, where A_control_ was the absorbance of the blank control and A_test_ was the absorbance of a test reaction.

The scavenging ability of purified violaxanthin against the ABTS^+^ radical was measured using a method by Chen et al. [34] with some modifications. The stock solutions of ABTS (7 mM) and potassium peroxydisulfate (2.45 mM) were firstly prepared with deionized water. One milliliter of ABTS solution and 1 mL of potassium peroxydisulfate solution were mixed and stored in the dark at room temperature for 12–16 h. Then, the mixed solution was diluted with ethanol to obtain an appropriate concentration of the ABTS^+^ working solution (A_734_ = 0.70). Two milliliters of the ABTS^+^ working solution was mixed with 1 mL of purified violaxanthin at concentrations ranging from 1.25–80 μg mL^−1^, and the solution was incubated at room temperature for 6 min. The absorbance at 734 nm was rapidly measured, and the scavenging ability was calculated as: ABTS radical scavenging activity (%) = [(A_control_ − A _test_)/A_control_] × 100, where A_control_ denotes the absorbance of the blank control, and A_test_ denotes the absorbance of the test reaction.

### 3.7. Statistical Analysis

All measurements were carried out at least in triplicate, and the experimental data are expressed as the mean ± SD (standard deviation). Student-Newman-Keul’s test using one-way ANOVA was performed using a statistical analysis software package (SPSS 19.0, IBM Corporation, Armonk, NY, USA), and statistically significant difference were *p* < 0.05.

## 4. Conclusions

This work revealed that light intensity and nitrogen concentration were the main factors affecting the accumulation of violaxanthin in *Eustigmatos* cf. *polyphem*. The fed-batch culture was may be more suitable for maintaining the vigorous growth and a high level of violaxanthin under low-light irradiation. The violaxanthin purified from *Eustigmatos* cf. *polyphem* shows a potent efficacy in the scavenging of DPPH and ABTS^+^ radicals, which indicates that violaxanthin could be used as a natural antioxidant agent for therapeutic or functional adjuvant purposes.

## Figures and Tables

**Table 1:** Production of biomass and violaxanthin in *Eustigmatos* cf. *polyphem* grown in batch culture and fed-batch culture under low light illumination.

Culture Mode	Time (Day)	Biomass (g/L)	Violaxanthin Content (% of DW)	Violaxanthin Productivity (mg/L/d)
**Batch culture**	5th	1.36 ± 0.03 ^a^	0.39 ± 0.01 ^a^	1.07 ± 0.03 ^a^
**(control)**	10th	2.53 ± 0.04 ^b^	0.32 ± 0.01 ^b^	0.82 ± 0.03 ^b^
	15th	3.39 ± 0.06 ^c^	0.27 ± 0.02 ^c^	0.62 ± 0.04 ^c^
	20th	4.37 ± 0.03 ^d^	0.23 ± 0.01 ^d^	0.51 ± 0.02 ^d^
**Fed-batch**	5th	1.36 ± 0.03 ^a^	0.39 ± 0.02 ^a^	1.07 ± 0.03 ^a^
**culture**	10th	2.92 ± 0.01 ^e^	0.38 ± 0.01 ^e^	1.10 ± 0.03 ^a^
	15th	4.02 ± 0.05 ^f^	0.36 ± 0.01 ^f^	0.96 ± 0.02 ^e^
	20th	5.15 ± 0.03 ^g^	0.35 ± 0.02 ^f^	0.90 ± 0.06 ^be^

DW, dry weight. Values are expressed as the mean ± SD from three replicates. Values with different superscript letters (a, b, c, d, e, f, and g) in the same column are significantly different (p < 0.05).

## References

[1] Rodriguez-Amaya D.B. (2001). A Guide to Carotenoid Analysis in Foods.

[2] Fu H., Xie B., Ma S., Zhu X., Fan G., Pan S. (2011). Evaluation of antioxidant activities of principal carotenoids available in water spinach (*Ipomoea aquatica*). J. Food. Compos. Anal..

[3] Liu D., Shi J., Ibarra A.C., Kakuda Y., Xue S.J. (2008). The scavenging capacity and synergistic effects of lycopene, vitamin E, vitamin C, and β-carotene mixtures on the DPPH free radical. LWT-Food Sci. Technol..

[4] Müller L., Fröhlich K., Böhm V. (2011). Comparative antioxidant activities of carotenoids measured by ferric reducing antioxidant power (FRAP), ABTS bleaching assay (αTEAC), DPPH assay and peroxyl radical scavenging assay. Food Chem..

[5] Fraser P.D., Bramley P.M. (2004). The biosynthesis and nutritional uses of carotenoids. Prog. Lipid Res..

[6] Milledge J.J. (2011). Commercial application of microalgae other than as biofuels: A brief review. Rev. Environ. Sci. Bio/Technol..

[7] Rajauria G., Foley B., Abu-Ghannam N. (2017). Characterization of dietary fucoxanthin from *Himanthalia elongata* brown seaweed. Food Res. Int..

[8] Cano M., Gómez-Maqueo A., García-Cayuela T., Welti-Chanes J. (2017). Characterization of carotenoid profile of Spanish *Sanguinos* and *Verdal* prickly pear (*Opuntia ficus-indica*, spp.) tissues. Food Chem..

[9] Hu C.C., Lin J.T., Lu F.J., Chou F.P., Yang D.J. (2008). Determination of carotenoids in *Dunaliella salina* cultivated in Taiwan and antioxidant capacity of the algal carotenoid extract. Food Chem..

[10] Soontornchaiboon W., Joo S.S., Kim S.M. (2012). Anti-inflammatory effects of violaxanthin isolated from microalga *Chlorella ellipsoidea* in RAW 264.7 macrophages. Biol. Pharm. Bull..

[11] Hagen C., Braune W., Vogel K., HÄDER D.P. (1993). Functional aspects of secondary carotenoids in *Haematococcus lacustris* (Girod) Rostafinski (Volvocales). V. Influences on photomovement. Plant Cell Environ..

[12] Guedes A.C., Amaro H.M., Malcata F.X. (2011). Microalgae as sources of carotenoids. Mar. Drugs.

[13] Meléndez-Martínez A.J., Britton G., Vicario I.M., Heredia F.J. (2008). The complex carotenoid pattern of orange juices from concentrate. Food Chem..

[14] Shukla M., Kumar S. (2018). Algal biorefineries for biofuels and other value-added products. Biorefining of Biomass to Biofuels.

[15] Pasquet V., Morisset P., Ihammouine S., Chepied A., Aumailley L., Berard J.-B., Serive B., Kaas R., Lanneluc I., Thiery V. (2011). Antiproliferative activity of violaxanthin isolated from bioguided fractionation of *Dunaliella tertiolecta* extracts. Mar. Drugs.

[16] Talero E., García-Mauriño S., Ávila-Román J., Rodríguez-Luna A., Alcaide A., Motilva V. (2015). Bioactive compounds isolated from microalgae in chronic inflammation and cancer. Mar. Drugs.

[17] Zhang J., Wan L., Xia S., Li A., Zhang C. (2013). Morphological and spectrometric analyses of lipids accumulation in a novel oleaginous microalga, *Eustigmatos* cf. *polyphem* (Eustigmatophyceae). Bioprocess Biosyst. Eng..

[18] Li Z., Ma X., Li A., Zhang C. (2012). A novel potential source of β-carotene: *Eustigmatos* cf. *polyphem* (Eustigmatophyceae) and pilot β-carotene production in bubble column and flat panel photobioreactors. Bioresour. Technol..

[19] Gao B., Xia S., Lei X., Zhang C. (2018). Combined effects of different nitrogen sources and levels and light intensities on growth and fatty acid and lipid production of oleaginous eustigmatophycean microalga *Eustigmatos* cf. polyphem. J. Appl. Phycol..

[20] Eliáš M., Amaral R., Fawley K.P., Fawley M.W., Němcová Y., Neustupa J., Přibyl P., Santos L.M., Ševčíková T. (2017). Handbook of the Protists.

[21] Keşan G., Litvín R., Bína D., Durchan M., Šlouf V., Polívka T. (2016). Efficient light-harvesting using non-carbonyl carotenoids: Energy transfer dynamics in the VCP complex from *Nannochloropsis oceanica*. BBA-Bioenerg..

[22] Llansola-Portoles M.J., Litvin R., Ilioaia C., Pascal A.A., Bina D., Robert B. (2017). Pigment structure in the violaxanthin–chlorophyll-a-binding protein VCP. Photosynth. Res..

[23] Bína D., Gardian Z., Herbstová M., Litvín R. (2017). Modular antenna of photosystem I in secondary plastids of red algal origin: A *Nannochloropsis oceanica* case study. Photosynth. Rese..

[24] Demmig-Adams B., Adams W.W. (1996). Xanthophyll cycle and light stress in nature: Uniform response to excess direct sunlight among higher plant species. Planta.

[25] Uhrmacher S., Hanelt D., Nultsch W. (1995). Zeaxanthin content and the degree of photoinhibition are linearly correlated in the brown alga *Dictyota dichotoma*. Mar. Biol..

[26] Gao B., Yang J., Lei X., Xia S., Li A., Zhang C. (2016). Characterization of cell structural change, growth, lipid accumulation, and pigment profile of a novel oleaginous microalga, *Vischeria stellata* (Eustigmatophyceae), cultured with different initial nitrate supplies. J. Appl. Phycol..

[27] Lubián L.M., Montero O., Moreno-Garrido I., Huertas I.E., Sobrino C., González-del Valle M., Parés G. (2000). *Nannochloropsis* (Eustigmatophyceae) as source of commercially valuable pigments. J. Appl. Phycol..

[28] Peccia J., Haznedaroglu B., Gutierrez J., Zimmerman J.B. (2013). Nitrogen supply is an important driver of sustainable microalgae biofuel production. Trends Biotechnol..

[29] Dong Y.R., Cheng S.J., Qi G.H., Yang Z.P., Yin S.Y., Chen G.T. (2017). Antimicrobial and antioxidant activities of *Flammulina velutipes* polysacchrides and polysacchride-iron (III) complex. Carbohydr. Polym..

[30] Sharma O.P., Bhat T.K. (2009). DPPH antioxidant assay revisited. Food Chem..

[31] Zhang J., Hou X., Ahmad H., Zhang H., Zhang L., Wang T. (2014). Assessment of free radicals scavenging activity of seven natural pigments and protective effects in AAPH-challenged chicken erythrocytes. Food Chem..

[32] Xia S., Wang K., Wan L., Li A., Hu Q., Zhang C. (2013). Production, characterization, and antioxidant activity of fucoxanthin from the marine diatom *Odontella aurita*. Mar. Drugs.

[33] Bai K., Xu W., Zhang J., Kou T., Niu Y., Wan X., Zhang L., Wang C., Wang T. (2016). Assessment of free radical scavenging activity of dimethylglycine sodium salt and its role in providing protection against lipopolysaccharide-induced oxidative stress in mice. PLoS ONE.

[34] Li X., Lin J., Gao Y., Han W., Chen D. (2012). Antioxidant activity and mechanism of Rhizoma *Cimicifugae*. Chem. Cent. J..

